# The Prognostic and Clinicopathological Significance of Tumor-Associated Macrophages in Patients with Gastric Cancer: A Meta-Analysis

**DOI:** 10.1371/journal.pone.0170042

**Published:** 2017-01-12

**Authors:** Songcheng Yin, Jinyu Huang, Zhan Li, Junyan Zhang, Jiazi Luo, Chunyang Lu, Hao Xu, Huimian Xu

**Affiliations:** 1 Department of Surgical Oncology, The First Hospital of China Medical University, Shenyang, Liaoning, China; 2 Department of Breast Surgery, The First Hospital of China Medical University, Shenyang, Liaoning, China; National Cancer Center, JAPAN

## Abstract

**Objective:**

Comprehensive studies have investigated the prognostic and clinicopathological value of tumor-associated macrophages (TAMs) in gastric cancer patients, yet results remain controversial. Therefore, we performed a meta-analysis to clarify this issue.

**Methods:**

PubMed, Embase, and the Cochrane Library databases were searched to identify eligible studies. We extracted hazard ratios (HRs) and odds ratios (ORs) with their corresponding 95% confidence intervals (95% CIs) to estimate the effect sizes. In addition, subgroup analysis and sensitivity analysis were also conducted.

**Results:**

A total of 19 studies involving 2242 patients were included. High generalised TAMs density was significantly associated with poor overall survival (OS) (HR 1.49, 95% CI 1.15–1.95). Subgroup analysis revealed that CD68^+^ TAMs had no significant effect on OS (HR 1.38, 95% CI 1.00–1.91). High M1 TAMs density was correlated with better OS (HR 0.45, 95% CI 0.32–0.65). By contrast, high density of M2 TAMs was correlated with a poor prognosis for OS (HR 1.48, 95% CI 1.25–1.75). Furthermore, high M2 TAMs density was correlated with larger tumor size, diffuse Lauren type, poor histologic differentiation, deeper tumor invasion, lymph node metastasis, and advanced TNM stage.

**Conclusions:**

Overall, this meta-analysis reveal that although CD68^+^ TAMs infiltration has the neutral prognostic effects on OS, the M1/M2 polarization of TAMs are predicative factor of prognosis in gastric cancer patients.

## Introduction

Gastric cancer represents the fifth most common malignancy and the third leading cause of cancer death in the world [1]. Despite recent advances in the diagnosis and medical treatment of gastric cancer, patient survival remains poor, especially for those in the advanced stages of the disease [2]. In addition, it has been reported that the current TNM classification scheme does not adequately reflect the tumor biological behavior and patient prognosis for gastric cancer [3]. Therefore, it is imperative to identify biomarkers to predict tumor progression and patient survival, as well as to provide novel therapeutic targets.

Tumor-associated macrophages (TAMs), as fundamental components of the inflammatory microenvironment of tumors, originate from circulating monocytes and are recruited to the tumor site [4, 5]. Different microenvironments can lead to two different polarizations of TAMs: the classically activated type M1 phenotype and the alternatively activated M2 phenotype. M1 macrophages are considered to be induced by Th1 cytokines (e.g., interferon-γ), microbial stimuli (e.g., lipopolysaccharide) and tumor necrosis factor α, with the function of promoting an inflammatory response and antitumor activity. M2 macrophages are mainly activated by Th2 cytokines (e.g., interleukin 4, interleukin 13), which participate in the anti-inflammatory response, tissue remodeling, angiogenesis and tumor cell activation [5–8].

The role of TAMs in the tumor microenvironment as well as their prognostic value have been widely discussed in many human cancers such as breast [9], lung [10], prostate [11], liver [12] and gastric cancer [13]. However, there exists controversy regarding the impact of TAMs on patient prognosis and clinicopathological characteristics of gastric cancer. Numerous publications have demonstrated that the TAMs density was associated with poor prognosis [13–15]; on the contrary, some studies hold different views [16–18]. Moreover, several articles reported that the polarizing subtypes of TAMs have different prognostic effects [19, 20]. To resolve these inconsistencies as well as to identify more precise prognostic biomarkers, we performed a meta-analysis to evaluate the correlation between TAMs density and its prognostic and clinicopathological significance in patients with gastric cancer.

## Materials and Methods

### Search strategy and selection criteria

A comprehensive literature search of PubMed, Embase, and the Cochrane Library databases was conducted from their inception through August 17, 2016. The following key words were variably combined: “stomach”, “gastric”, “neoplasm”, “cancer”, “carcinoma”, “tumor”, “macrophage”, “tumor-associated macrophage”, and “tumor-infiltrating macrophage”. Additionally, we also manually checked the reference entries of the relevant literature to minimize any omissions that may have occurred during the search process. This meta-analysis was based on previously published articles; therefore, ethical approval was not required.

To identify eligible studies, the inclusion criteria for this meta-analysis was established as follows: (1) gastric cancer as the target disease, (2) detected macrophage density in primary tumor tissues, (3) correlation of macrophage density with either prognosis (e.g., OS, DFS) or clinicopathological characteristics, (4) sufficient data to extract hazard ratios (HRs), the odds ratio (ORs), and their 95% confidence intervals (CIs), and (5) full text publications in English. Specific types of literature such as reviews, comments and conference abstracts were not included in our meta-analysis. If overlapping patients were reported with different TAMs markers or distribution among the articles, all of the reported incidents were included for different objective analysis. The process of the literature search was independently finished by two authors (Songcheng Yin and Jinyu Huang).

### Quality assessment

The Newcastle-Ottawa scale (NOS) [21] was used to evaluate the quality of the original studies. This scale mainly involves three components: patient selection, study comparability and outcome assessment. Each of the included studies obtained a score between 0 and 9. Studies with an NOS score ≥6 were regarded as high quality. Two authors (Songcheng Yin and Zhan Li) independently performed this assessment, and discrepancies were resolved by discussion.

### Data extraction

Two reviewers (Songcheng Yin and Jiazi Luo) independently performed the data extraction. The relevant data from the included studies comprised the first author’s name, publication year, country or area, number of patients, age, gender, makers of macrophage, detection methods of macrophage density, cut-off value, clinicopathological parameters, and survival data. Any inconsistencies were resolved through negotiation and consultation.

### Statistical analysis

The hazard ratios (HRs) with 95% confidence intervals (CIs) were applied to investigate the association between the TAMs density and survival of patients with gastric cancer. For time-to-event outcomes, HRs and their 95% CIs were given directly in most of the original studies. However, several articles presented Kaplan–Meier curves rather than the HR; therefore, the HR was calculated from the survival curves using the methods reported by Parmar and Tierney [22, 23]. Odds ratios (ORs) with confidence intervals (CIs) were used to evaluate the correlation between the TAMs density and clinicopathological characteristics. A combined HR and OR >1 suggested a worse prognosis in the high TAMs density group and was regarded to be statistically significant if the 95% CI did not overlap 1.

Heterogeneity among the studies was assessed by the Cochran’s Q statistic and *I*^*2*^ tests [24]. Either *P*<0.10 or *I*^*2*^ statistic >50% defined significant heterogeneity across the articles, in which case the random effects model was performed; otherwise, the fixed effects model was implemented. To find the source of heterogeneity and assess its effect on the outcome of various variables, a subgroup analysis was conducted. In addition, whether the combined results were stable, we performed a sensitivity analysis to gauge this stability. Meanwhile, Begg’s test [25] and Egger’s test [26] regression model were used to test for publication bias. All statistical analysis programs were performed using STATA version 12.0 (Stata, College Station, TX, USA), and all *P* values were two-sided.

## Results

### Search results

**Fig 1:**
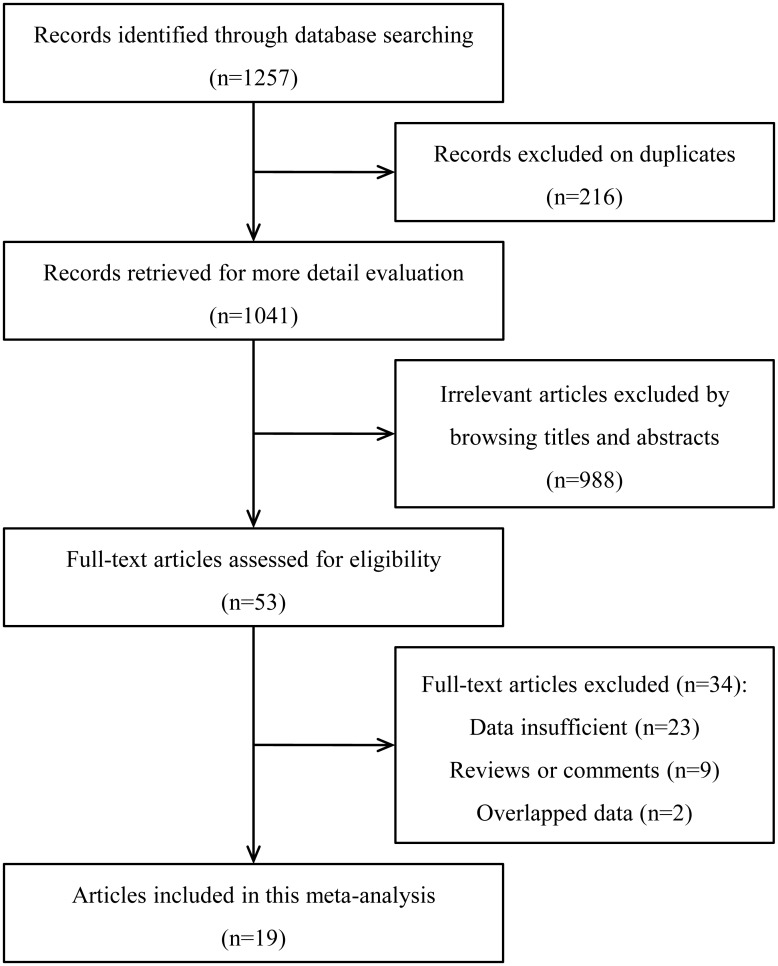
Flow chart of the study selection process.

A total of 1257 articles were initially identified in our systematic literature search. Following the exclusion of duplicate publications, 1041 records remained. After screening the titles and abstracts, another 988 articles were excluded. Then, we systematically reviewed the remaining full text articles and precluded another 34 studies because of inconsistencies with the selection criteria. Finally, 19 articles [13–20, 27–37] published between 2003 and 2016 were included in this meta-analysis (Fig 1).

### Study characteristics

Among the included studies, different markers (CD68, CD163, CD204, etc.) were used to label the TAMs types and their localized distribution (e.g., intratumor and stroma). Some participants were enrolled twice for marker-specific or distribution-specific analyses in different articles [13, 28, 36, 37]. Therefore, 2242 gastric cancer patients were included in this meta-analysis. CD68^+^ was used as an ordinary maker of TAMs in 15 articles. M1 TAMs (labeled by CD11c, and NOS2) and M2 TAMs (labeled by CD163, CD204, and CD206) were reported in 2 and 9 articles, respectively. The fundamental features and study quality of the 19 eligible studies are summarized in Table 1.

**Table 1:** Characteristics of studies included in the meta-analysis.

Study	Year	Region	Cases	Stage	Makers	Methods	Cut-off	Outcome	NOS
Zhang [13]	2016	China	178	I–IV	CD68	IHC	Score ≥6	OS	7
Kim [27]	2016	Korea	396	I–IV	CD68/CD163	IHC	NR	OS	6
Yan [28]	2016	China	178	I–IV	CD163	IHC	Score ≥6	OS	8
Ichimura [29]	2016	Japan	119	I–III	CD204	IHC	Density ≥0.22%	OS	7
Park [30]	2016	Korea	113	I–IV	CD163	IHC	Density ≥77%	OS/DFS	8
Ding [31]	2016	China	48	I–IV	CD68	IHC	NR	-	6
Lin [17]	2015	Taiwan	170	Early/ Advanced	CD204	IHC	Intensity >50%	OS	6
Zhang [19]	2015	China	180	I–IV	CD68/CD11c/CD206	IHC	Density	OS	8
Kim [32]	2015	Korea	143	I–III	CD68/CD163	IHC	Score ≥1	DFS	6
Wu [15]	2015	Taiwan	103	I–IV	CD68	IHC	≥671 cells/HPF	OS	9
Pantano [20]	2013	Italy	52	I–III	CD68+NOS2/CD68+CD163	IF	Median score	OS	9
Peng [33]	2012	China	184	I–IV	NR	IF	Density >20%	OS	6
Osinsky [16]	2011	Ukraine	105	I–IV	CD68	IHC	Density >23%	OS	6
Wang [18]	2011	China	107	T2–T3	CD68	IHC	>67.2 cells/HPF	OS	8
Kawahara [34]	2010	Japan	111	I–IV	CD68/CD163	IHC	NR	OS	7
Haas [35]	2009	Germany	52	I–IV	CD68	IHC	Median density	DFS	8
Ohno [36]	2005	Japan	84	T2–T3	CD68	IHC	Density ≥21.4%	DFS	9
Ishigami [14]	2003	Japan	97	I–IV	CD68	IHC	≥200 cells/HPF	OS	7
Ohno [37]	2003	Japan	84	T2–T3	CD68	IHC	Density ≥4.7%	DFS	9

IHC, immunohistochemistry; IF, immunofluorescence; OS, overall survival; DFS, disease-free survival; NOS, Newcastle-Ottawa Scale; HPF, high-power fields; NR, not reported.

### TAMs density and OS in gastric cancer patients

**Fig 2:**
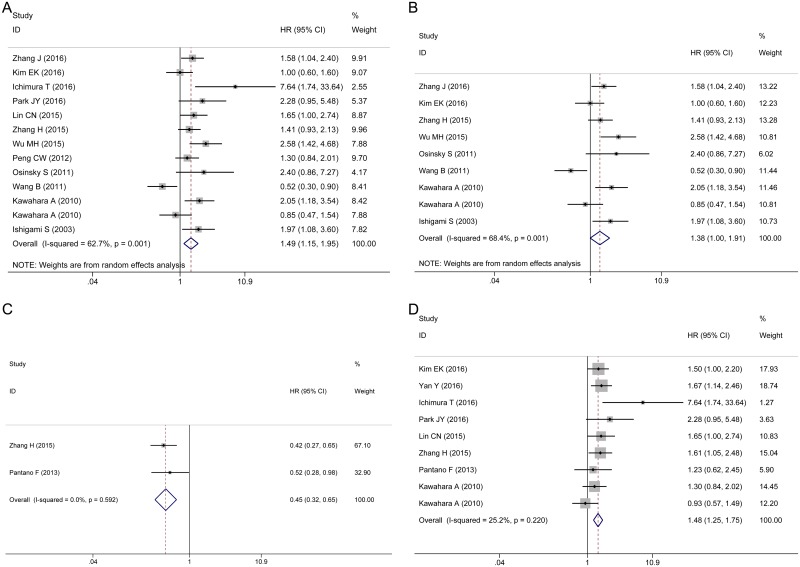
Forest plots of HRs for the correlation between TAMs density and OS. (A) Generalized TAMs. (B) CD68^+^ TAMs. (C) M1 TAMs. (D) M2 TAMs.

There is a phenomenon that multiple markers were used to estimate the impact of TAMs density on patient survival. When CD68 and other marker data were reported in a single study, we choose CD68, a common macrophage marker, as the indicator of TAMs detection to prevent the incorporation of duplicate samples. The pooled HR showed that a highly generalized TAMs density was significantly associated with poor OS (HR 1.49, 95% CI 1.15–1.95, Fig 2A). Due to the presence of significant heterogeneity among the studies (*I*^*2*^ = 62.7%, *P* = 0.001), the random effects model was adopted. Among the 12 studies, 11 studies estimated the TAMs density in both the intratumor and stroma regions. While the study by Park [30] respectively assessed intratumoral and stromal TAMs density, we extracted only the intratumoral data. However, the results did not change after removing this study (HR 1.46, 95% CI 1.11–1.92).

In view of the variety of markers used to detect TAMs density and the existence of substantial heterogeneity, we conducted a subgroup analysis according to the different markers and polarizations. We examined the effect of CD68^+^, M1 and M2 TAMs on the overall survival of patients with gastric cancer. There was no significant association between the CD68^+^ TAMs density and OS (HR 1.38, 95% CI 1.00–1.91, *P* = 0.052, *I*^*2*^ = 68.4%, *P* = 0.001, random effects model, Fig 2B). A high M1 TAMs density was correlated with better OS (HR 0.45, 95% CI 0.32–0.65, *P*<0.001, *I*^*2*^ = 0%, *P* = 0.592, fixed effects model, Fig 2C). Nevertheless, a high density of M2 TAMs was correlated with a poor prognosis for OS (HR 1.48, 95% CI 1.25–1.75, *P*<0.001, *I*^*2*^ = 25.2%, *P* = 0.22, fixed effects model, Fig 2D).

### TAMs density and DFS in gastric cancer patients

Several studies provided data concerning the association between TAMs infiltration and DFS stratified by different TAMs markers (CD68^+^ and M2 TAMs) and localized distribution (intratumor and stroma).

**Fig 3:**
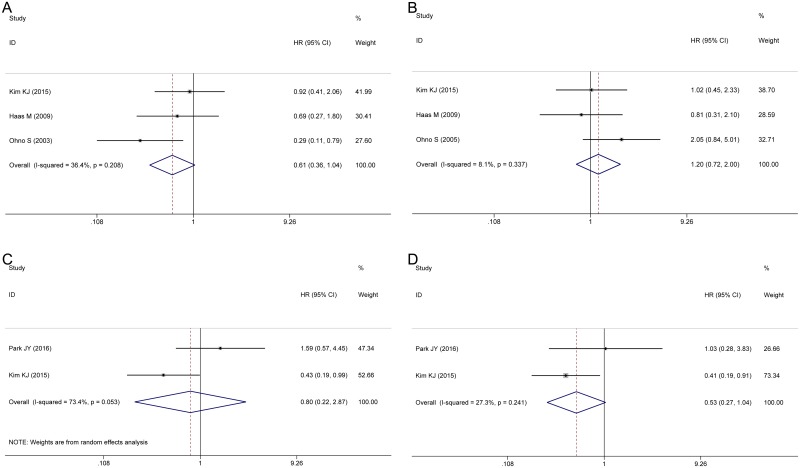
Forest plots of HRs for the correlation between TAMs density and DFS. (A) Intratumor CD68^+^ TAMs. (B) Stroma CD68^+^ TAMs. (C) Intratumor M2 TAMs. (D) Stroma M2 TAMs.

The pooled HRs from three studies indicated that there was no association between the CD68^+^ TAMs density and DFS in either the intratumor (HR 0.61, 95% CI 0.36–1.04, *P* = 0.068, *I*^*2*^ = 36.4%, *P* = 0.208, Fig 3A) or stroma (HR 1.20, 95% CI 0.72–2.00, *P* = 0.458, *I*^*2*^ = 8.1%, *P* = 0.337, Fig 3B). Two studies estimated the correlation between M2 TAMs and DFS in the intratumor and stroma (Fig 3C and 3D). The pooled HRs showed that the density of M2 TAMs was not correlated with DFS in either the intratumor (HR 0.80, 95% CI 0.22–2.87, *P* = 0.728, *I*^*2*^ = 73.4%, *P* = 0.053) or stroma (HR 0.53, 95% CI 0.27–1.04, *P* = 0.063, *I*^*2*^ = 27.3%, *P* = 0.241). Due to the limited number of articles, one should be cautious regarding the interpretation of these results.

### TAMs density and clinicopathological features

To further elucidate the role of TAMs infiltration on tumor progression, we investigated the correlation between TAMs density and the clinicopathological features of gastric cancer according to different markers and polarizations. Because only one study reported M1 TAMs data, we focused on CD68^+^ TAMs and M2 TAMs. All of the studies describing the patients’ clinicopathological characteristics detected TAMs in both the intratumor and stroma except the study by Park [30], in which the intratumoral data were used.

As shown in Table 2, the CD68^+^ TAMs density was not associated with age (older vs. younger: OR 0.83, 95% CI 0.56–1.24, *P* = 0.365), gender (male vs. female: OR 0.73, 95% CI 0.53–1.00, *P* = 0.051), tumor size (large vs. small: OR 1.27, 95% CI 0.89–1.80, *P* = 0.185), depth of invasion (T3–T4 vs. T1–T2: OR 1.54, 95% CI 0.90–2.63, *P* = 0.112), lymph node metastasis (present vs. absent: OR 1.67, 95% CI 0.92–3.03, *P* = 0.093), or Lauren type (diffuse vs. intestinal: OR 1.49, 95% CI 0.77–2.88, *P* = 0.232). However, a high density of CD68^+^ TAMs was significantly correlated with advanced TNM stage (III–IV vs. I–II: OR 2.61, 95% CI 1.82–2.73, *P*<0.001) and poor histological differentiation (poorly vs. well to moderately: OR 1.73, 95% CI 1.00–2.98, *P* = 0.048).

**Table 2:** The relationship between TAMs and clinicopathological characteristics.

Clinicopathological features	No. of studies	Pooled OR	*P* value	Heterogeneity		Effect model	Publication bias	
		(95% CI)		*I*^*2*^ (%)	*P* value		*P*_*Egger*_	*P*_*Begg*_
**CD68**^**+**^ **TAMs**								
Age	4	0.83 (0.56–1.24)	0.365	6.4	0.361	Fixed	0.590	0.734
Gender	6	0.73 (0.53–1.00)	0.051	0.0	0.791	Fixed	0.564	0.452
Tumor size	4	1.27 (0.89–1.80)	0.185	45.1	0.141	Fixed	0.740	1.000
Lauren classifcation	4	1.49 (0.77–2.88)	0.232	63.9	0.040	Random	0.489	0.308
Grade of differentiation	5	1.73 (1.00–2.98)	0.048	53.9	0.069	Random	0.903	1.000
Depth of invasion	6	1.54 (0.90–2.63)	0.112	50.2	0.074	Random	0.090	0.707
Lymph node metastasis	7	1.67 (0.92–3.03)	0.093	69.0	0.004	Random	0.735	1.000
TNM stage	5	2.61 (1.82–3.73)	<0.001	31.4	0.212	Fixed	0.333	0.462
**M2 TAMs**								
Age	5	1.09 (0.60–1.97)	0.780	66.1	0.019	Random	0.821	0.806
Gender	6	0.79 (0.59–1.06)	0.117	0.0	0.453	Fixed	0.232	0.707
Tumor size	4	1.61 (1.17–2.23)	0.004	46.2	0.134	Fixed	0.530	0.734
Lauren classifcation	5	1.52 (1.10–2.11)	0.012	23.7	0.264	Fixed	0.315	0.462
Grade of differentiation	4	2.78 (1.94–3.97)	<0.001	16.9	0.307	Fixed	0.457	0.734
Depth of invasion	5	2.56 (1.24–5.28)	0.011	75.2	0.003	Random	0.453	1.000
Lymph node metastasis	6	2.17 (1.40–3.38)	0.001	52.9	0.059	Random	0.788	1.000
TNM stage	6	2.26 (1.32–3.87)	0.003	67.5	0.009	Random	0.828	1.000

In addition, the pooled analysis revealed no significant association between M2 TAMs and age (older vs. younger: OR 1.09, 95% CI 0.60–1.97, *P* = 0.780) or gender (male vs. female: OR 0.79, 95% CI 0.59–1.06, *P* = 0.117). Nevertheless, a high M2 TAMs density was correlated with several clinical parameters, including tumor size (large vs. small: OR 1.61, 95% CI 1.17–2.23, *P* = 0.004), depth of invasion (T3–T4 vs. T1–T2: OR 2.56, 95% CI 1.24–5.28, *P* = 0.011), lymph node metastasis (present vs. absent: OR 2.17, 95% CI 1.40–3.38, *P* = 0.001), TNM stage (III–IV vs. I–II: OR 2.26, 95% CI 1.32–3.87, *P* = 0.003), Lauren type (diffuse vs. intestinal: OR 1.52, 95% CI 1.10–2.11, *P* = 0.012), and histological differentiation (poorly vs. well to moderately: OR 2.78, 95% CI 1.94–3.97, *P*<0.001) (Table 2).

### Sensitivity analysis

**Fig 4:**
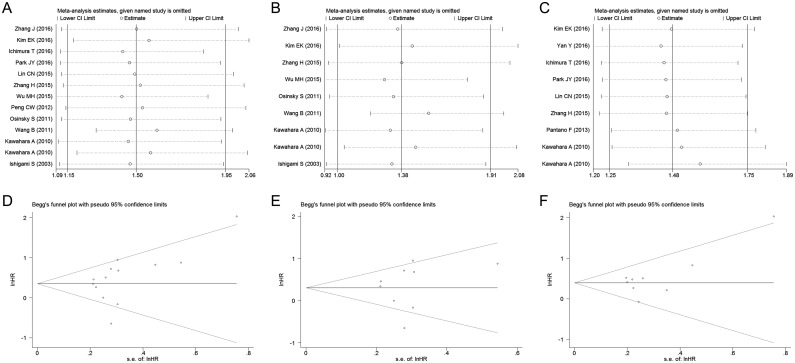
Sensitivity analysis of TAMs on OS and funnel plots of publication bias in analysis of OS. (A) Sensitivity analysis of generalised TAMs. (B) Sensitivity analysis of CD68^+^ TAMs. (C) Sensitivity analysis of M2 TAMs. (D) Funnel plot for generalised TAMs on OS. (E) Funnel plot for CD68^+^ TAMs. (F) Funnel plot for M2 TAMs.

We conducted a sensitivity analysis by removing each individual study to evaluate the effect of individual datasets on the pooled HRs and ORs. The results shown in Fig 4A–4C indicated that the pooled HRs for OS was not substantially changed. Similarly, our findings of the pooled ORs for the clinicopathological characteristics were also robust (S1 and S2 Figs).

### Publication bias

Both Begg’s test and Egger’s test were applied to estimate the publication bias. The *P* values from these two tests suggested no evidence of publication bias on TAMs and OS (generalized TAMs: *P*_*Begg*_ = 0.127, *P*_*Egger*_ = 0.152, Fig 4D; CD68^+^ TAMs: *P*_*Begg*_ = 0.602, *P*_*Egger*_ = 0.686, Fig 4E; M2 TAMs: *P*_*Begg*_ = 0.466, *P*_*Egger*_ = 0.145, Fig 4F). The publication bias of M1 TAMs on OS and TAMs on DFS were not performed because of the small number of available articles. Moreover, all of the instances of *P*>0.05 (Begg’s test and Egger’s test) indicated that the assessment of publication bias was not significant in analysis of the clinicopathological features (Table 2).

## Discussion

It is well known that Hanahan and Weinberg emphasized the central role of tumor cells in tumor progression based on changes in the intracellular signaling of the tumor [38]. After a decade, they published another important review to further propose the key role of the tumor microenvironment in the occurrence and development of tumors [39]. The tumor microenvironment is mainly composed of tumor cells, immune cells, the extracellular matrix, and cytokines [40]. Recently, immunotherapy targeting immunosuppressive proteins such as cytotoxic T-lymphocyte associated protein 4 (CTLA-4) and programmed cell death 1 (PD-1) has provided more treatment options for cancer patients [41, 42]. Because the composition of the tumor microenvironment and the interactions among the molecules within this setting are complex, exploring novel targets for combination therapy is still crucial. TAMs, as important members of the tumor microenvironment, have two polarization subtypes: M1 and M2. M1 TAMs function by combating pathogens and tumor cells [7, 43]. In contrast, M2 TAMs are involved in promoting tumor progression. M2 TAMs not only inhibit immune response by producing TGF-β and IL-10 but also produce a variety of enzymes to degrade the matrix, which promotes the dissolution of the matrix membrane, interstitial digestion and remodeling. Moreover, they produce cytokines (e.g., VEGF, PDGF) that participate in angiogenesis and lymphatic vessel formation. Therefore, TAMs themselves and their polarization mechanism are regarded as novel therapeutic targets for cancer patients [4, 7, 44].

Indeed, the density of TAMs was found to be involved in the prognosis of various cancers. Nonomura suggested that a higher TAMs density correlated with poor recurrence-free survival in patients with prostate cancer [11]. Mei reported that high levels of M1 or M2 were associated with good or poor survival, respectively, in patients with non-small cell lung cancer [45]. However, there are still inconsistent prognostic data of TAMs and their polarization subtypes in gastric cancer. Therefore, we performed this meta-analysis to evaluate the prognostic impact and clinicopathological significance of TAMs in patients with gastric cancer.

We primarily assessed the association between generalized TAMs density and overall survival. The overall analysis showed that high density of generalized TAMs predicts a poor OS. Subgroup analysis indicated that the CD68^+^ TAMs density had no significant association with OS. However, a high M1 TAMs density was significantly correlated with better OS. In contrast, a high density of M2 TAMs was significantly correlated with a poor OS. It is well known that TAMs are a heterogeneous group of immune cells. The M1 and M2 phenotypes are more accurate descriptors of TAMs after polarization. A subgroup analysis revealed completely different significantly prognostic effects of both phenotypes, which was consistent with the function of M1 and M2 TAMs regarding anti- and pro-tumor progression, respectively. However, CD68 is a common marker that identifies both M1 and M2 TAMs and cannot reflect the TAMs polarization subtypes. The reason that CD68^+^ TAMs are not reliable as a prognostic marker in our analysis might be because of the neutralization of the M1 and M2 prognostic effects. Taking this into account, the relationship between CD68 and OS was not critically significant (*P* = 0.052). CD68 as a common marker of TAMs, whose relationship with OS was inconsistent with the result of generalized TAMs. Therefore, the result that high generalized TAMs density was associated with OS might be not robust. However, the M1 or M2 TAMs are predicative factor of prognosis in gastric cancer patients.

Moreover, neither CD68^+^ TAMs nor M2 TAMs was associated with DFS. However, these results were merely derived from three studies and two studies, respectively. We anticipate further research in this area to evaluate the relationship between TAMs and DFS. In the aspect of the analysis of clinicopathological features, a high CD68^+^ TAMs density was associated with an advanced TNM stage and poor histological differentiation. A high M2 TAMs density was correlated with larger tumor size, deeper tumor invasion, lymph node metastasis, advanced TNM stage, diffuse Lauren type, and poor histological differentiation. In general, a high density of M2 TAMs can signify poor clinicopathological characteristics in patients with gastric cancer.

The tumor promoting activity observed by TAMs can be attributed to the function of M2 TAMs [46–48]. Many researchers have reported the characteristics of M2 TAMs in the tumor progression of different malignant tumors. Intraperitoneal TAMs that polarized towards the M2 phenotype facilitated peritoneal dissemination in gastric cancer by introducing peritoneal mesothelial cell injury and promoting tumor cell proliferation [49, 50]. Zhang et al. reported that M2 TAMs displayed the ability to induce the expression of VEGF-C in Lewis lung carcinoma cells and to increase lymphangiogenesis [51]. In human basal cell carcinoma (BCC), M2 TAMs enhanced the potential of invasion and angiogenesis through a COX-2-dependent pathway, resulting in the elevated release of VEGF-A, bFGF and MMP-9 from BCC cells [52]. In our meta-analysis, we also observed the poor prognostic impact of M2 TAMs in gastric cancer. In addition, corosolic acid, a triterpenoid compound, has been shown to significantly inhibit macrophage polarization into the M2 phenotype and suppress subcutaneous tumor development and lung metastasis in a murine cancer model [7]. As a result, targeted therapy based on this mechanism has the potential to be applied clinically, and accordingly, gastric cancer patients with a high M2 TAMs density may obtain a survival benefit from this approach.

Previously, there were two meta-analysis involving the density of TAMs and the prognosis of gastric cancer. Zhang et al. reported that TAMs had a negative effect on OS in gastric cancer, but only 5 studies were included in this analysis [53]. The other publication by Liu et al. summarized from 11 studies and showed no association between TAMs and OS in gastric cancer [54]. There was a clear inconsistency between the results of these two publications. To clarify this confusion, we conducted this meta-analysis. Comparing with the previous meta-analyses, we used a broad search strategy to systematically search electronic databases and manually scanned reference entries of relevant literature. As a result, the 19 eligible studies included in our meta-analysis. Our original goal was to assess the prognostic value of generalized TAMs density in gastric cancer patients. Nevertheless, taking into account the complexity of TAMs, it might be not reliable to evaluate the relationship between generalized TAMs and OS simply. Thus, we performed stratified analysis according to the polarization of TAMs, which was not conducted in previous articles. And the results showed that completely different significantly prognostic effects of M1 and M2 TAMs, respectively. The inconsistence of previous meta-analysis might be due to huge heterogeneity of TAMs. We performed specifically concentrate on the prognostic value of the TAMs subtypes, which has made our meta-analysis more constructive and advisable.

However, some limitations exist in our study. First, there was significant heterogeneity among the analysis of CD68^+^ TAMs on OS and TAMs on clinicopathological features. Nevertheless, it is well known that heterogeneity among the studies exists when conducting meta-analysis of observational studies [55, 56]. In our meta-analysis it might be derived from the differences in sample size, demographic data, tumor location, EBV status and experimental technique. We adopted a more conservative approach and used the random effects model if there was significant heterogeneity. Second, the included studies used different types of antibodies or dilution ratios even if detecting the same TAMs marker. In addition, there was no international unification cutoff value to identify the density of the TAMs. Third, the number of the included studies was not enough to analyze the prognostic role of M1 TAMs, which was in turn weakened the power of the results.

In summary, our findings reveal that although CD68^+^ TAMs infiltration has the neutral prognostic effects on OS, the M1/M2 polarization of TAMs are predicative factor of prognosis in gastric cancer patients. Additional well-designed studies, especially multicenter and randomized controlled trials, are warranted to confirm our results and would provide more valuable prognostic information for gastric cancer patients.

## Supporting Information

S1 File Completed 2009 PRISMA Checklist. (DOC)

S1 Fig Sensitivity analysis for pooled ORs evaluating CD68+ TAMs density on clinicopathological features. (A) Patient's age. (B) Gender. (C) Tumor size. (D) Lauren classification. (E) Grade of differentiation. (F) Depth of invasion. (G) Lymph node metastasis. (H) TNM stage.
(TIF)

S2 Fig Sensitivity analysis for pooled ORs evaluating M2 TAMs density on clinicopathological features. (A) Patient's age. (B) Gender. (C) Tumor size. (D) Lauren classification. (E) Grade of differentiation. (F) Depth of invasion. (G) Lymph node metastasis. (H) TNM stage.
(TIF)
