# Supplementary figures and images for: The Prognostic and Clinicopathological Significance of Tumor-Associated Macrophages in Patients with Gastric Cancer: A Meta-Analysis
Source: PLoS One. 2017 Jan 12;12(1):e0170042. doi: 10.1371/journal.pone.0170042 (PMC5230964; doi:10.1371/journal.pone.0170042)

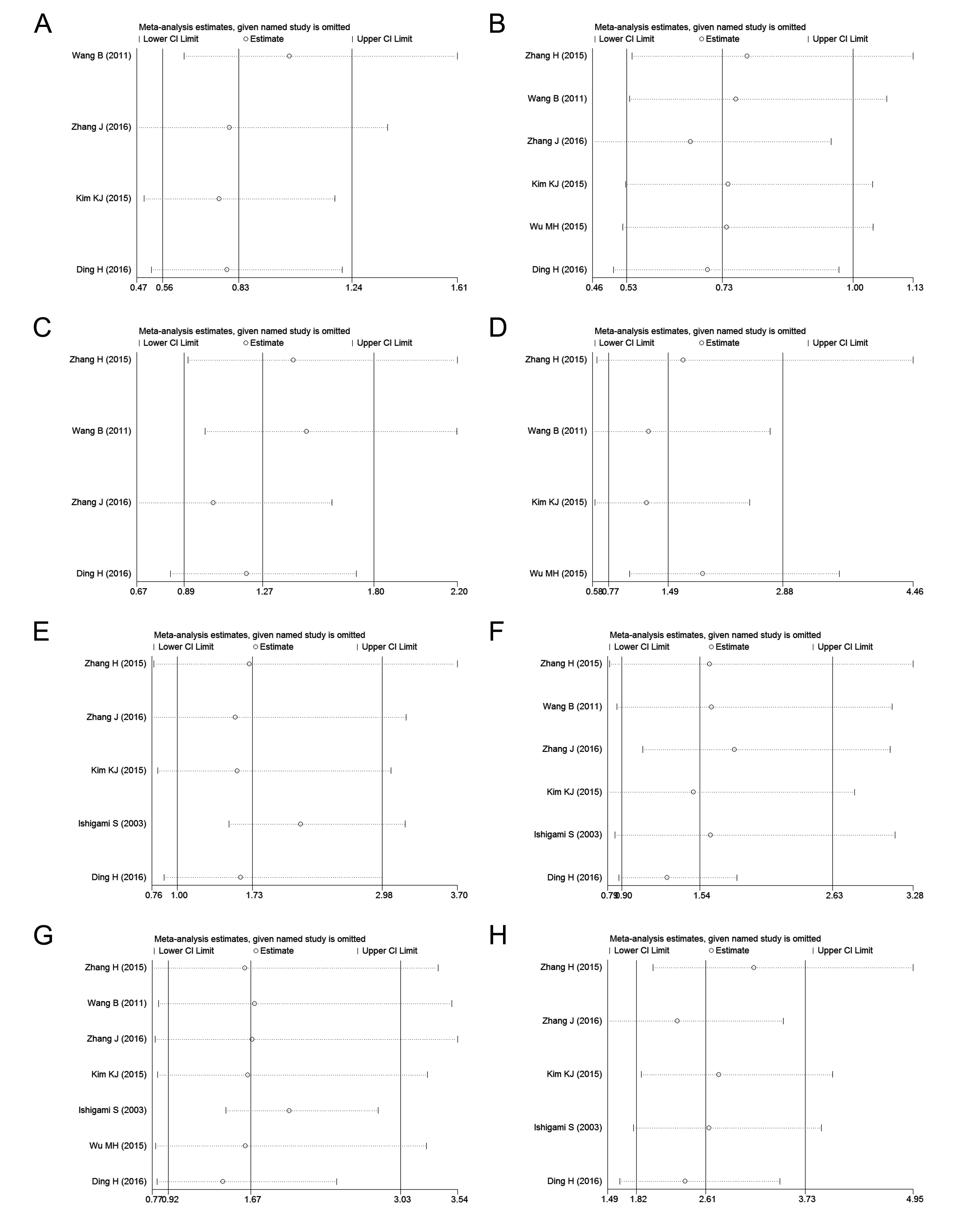

Supplement: S1 Fig — (A) Patient's age. (B) Gender. (C) Tumor size. (D) Lauren classification. (E) Grade of differentiation. (F) Depth of invasion. (G) Lymph node metastasis. (H) TNM stage. (TIF) [file pone.0170042.s002.tif]

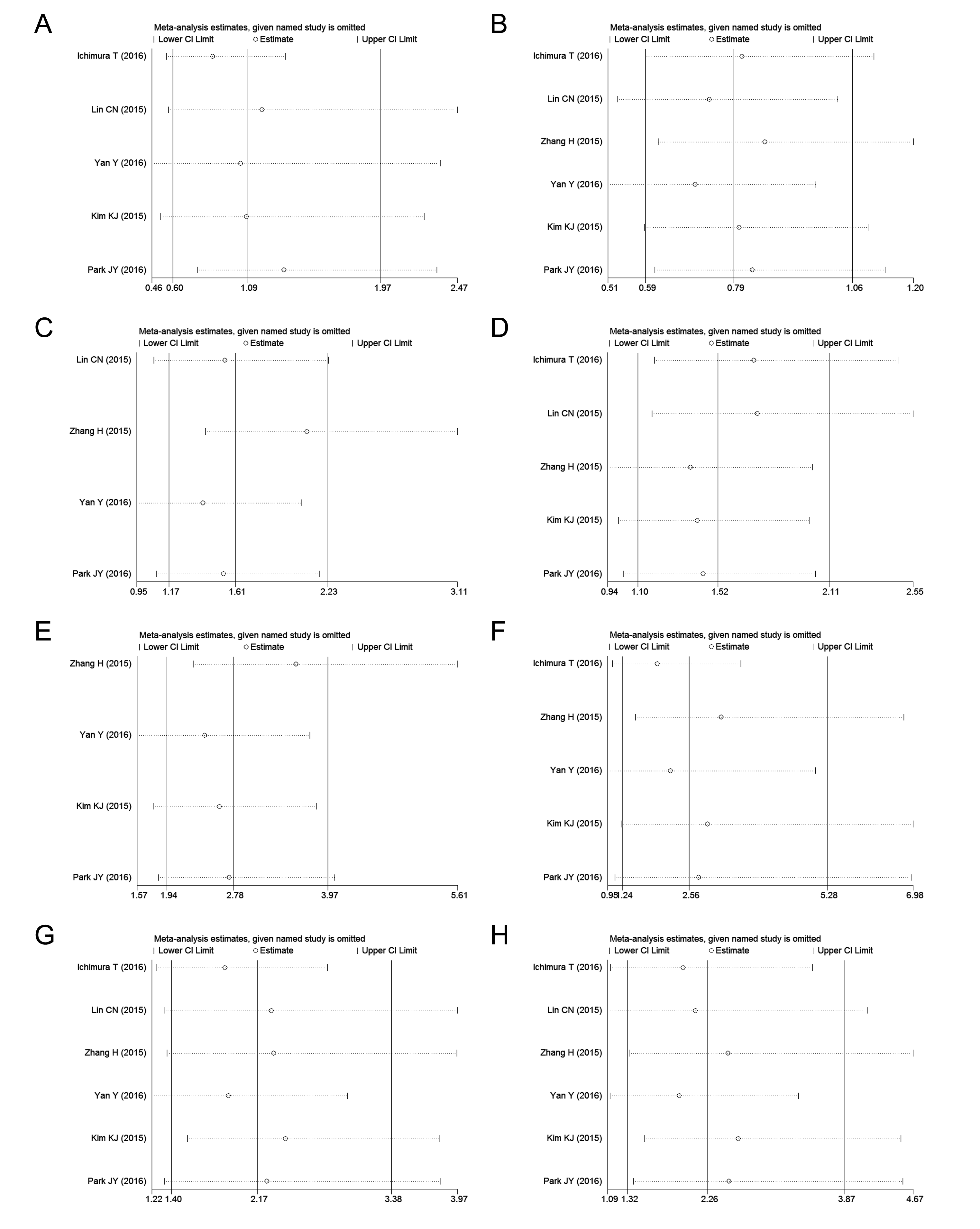

Supplement: S2 Fig — (A) Patient's age. (B) Gender. (C) Tumor size. (D) Lauren classification. (E) Grade of differentiation. (F) Depth of invasion. (G) Lymph node metastasis. (H) TNM stage. (TIF) [file pone.0170042.s003.tif]
